# 

**Published:** 1911-09-23

**Authors:** 


The jubilee of the *Revue Médicale*, one of the oldest weekly medical journals of Europe, has recently been celebrated at Cracow, the ancient metropolis and residence of the Kings of Poland. This journal is edited in its own offices by the Medical Society of Cracow, and is published gratis. All the Medical Chambers of Poland and of the other Slav nations were represented at the gathering, as well as many of the more important European medical journals.

The Prince of Monaco has just announced the creation of an Institute of Human Palæontology, which he has endowed with the revenue derived from a capital sum of 1,600,000 francs (£64,000), to be applied to the payment of salaries and expenses of research and publications. The Council of the Institute consists of M. Paul Distère, President of the section at the Council of State; M. Solomon Reinach, of the Institute; MM. Boule and Verneau, professors at the Museum of Natural History; M. Ernest Mayer, Maître des requêtes at the Council of State; and M. Louis Mayer, Privy Councillor to the Prince. M. Boule is Director of the Institute, and its two professors are MM. Breuil and Obermaier.

The death is announced from Rheims of Dr. Bruandet, Professor of Anatomy at the School of Medicine of that town. The unfortunate professor was the victim of duty, dying as the result of contracting anthrax from a patient whom he was attending.

### GIFTS.

Mr. Edward Snape Grigson, of Felixstowe, has left £2,000 to King Edward's Hospital Fund.

Mr. William Henry Herbert, of Coventry, has left £100 to the Coventry and Warwickshire Hospital.

Lieutenant-General Mostyn de la Poer Beresford has left £1,000 to the Free Home for the Dying, Clapham Common.

Mr. Abraham Greenwood Eastwood, of Todmorden, Yorks, has left £1,000 to the Royal Halifax Infirmary to endow a bed in the children's ward.

Mrs. Fanny Cleary, of North Walsham, Norfolk, has left £1,000 to the Norfolk and Norwich Hospital, and £500 to the Jenny Lind Hospital, Norwich.

The executors of Mr. Ralph Slazenger have allocated to the City of London Truss Society £100 out of the money left to be distributed at their discretion.

Mr. Ebenezer Hall, of Sheffield, has bequeathed £500 to the Sheffield Royal Hospital, £300 to the Sheffield Royal Infirmary, and £250 each to the Children's Hospital, Sheffield, the Wirksworth Hospital, and the Jessop Hospital for Women, Sheffield; and £100 to the Sheffield Nurses' Home.

Mr. J. E. Yonge, of Brixton, has bequeathed £500 to the South Devon and East Cornwall Hospital, Plymouth, and £250 each to the following institutions in the Plymouth district: The Royal Eye Infirmary, the Public Dispensary, the Ear and Throat Hospital, and the St. Elizabeth Convalescent Home.

Mr. Johann J. M. Hermann Spies has bequeathed the ultimate residue of his property, to the value of about £6,000, with contingent remainder, equally between the Hospital for Women, Soho Square, the National Hospital for Diseases of the Heart, the London Hospital, the German Hospital, the two Cottage Hospitals in Wimbledon, and three other charitable institutions.

Under the will of the late Mr. James Stedman Dixon, of Glasgow, the residue of his property, after the distribution of certain legacies and subject to certain conditions, is to be so divided that one-sixth will pass to each of the following Glasgow hospitals—namely, The Royal Infirmary, the Western Infirmary, and the Victoria Infirmary—while one-twelfth will go to the Glasgow Sick Children's Hospital.

### BUILDINGS CONTEMPLATED AND IN PROGRESS.

Homerton.—The Hackney Board of Guardians have accepted a tender of £20,342 for an extension to the Infirmary adjoining the Workhouse. The works comprise lunacy wards and Nurses' Home containing one hundred bedrooms. Mr. W. A. Finch, of London, is the architect.

Ealing.—From the designs of Messrs. Hall Jones and Cummings, already referred to in The Hospital, there has been erected at Ealing a King Edward Memorial Hospital at a cost of £17,000. It consists of an eighteen-bed pavilion on each of two floors, which is axially north and south, and one single-bed ward is placed on each floor, with the usual offices. Provision is made for a future pavilion and the operating theatre, which has been built, is placed contiguous to both, connected by glazed corridors. The administrative block is of three storeys and placed centrally between the pavilion block and the dispensary. To the south is placed a small casualty ward and the kitchen block; the south wall of the dispensary faces the mortuary block.

Lyons.—The old Hotel Dieu, founded in the sixth century, is to be superseded by a new hospital occupying some forty acres, at an approximate cost of £200,000. Special extensive laboratories are planned adjoining the university clinics, and a section for contagious diseases, with individual isolation, will be on the model of the Pasteur Hospital. Accommodation will be provided for 1,300 beds. A special commission of architects, physicians, and surgeons are considering the details, the aim being the provision of a thoroughly up-to-date institution.

Oxo was the only fluid beef which obtained the much-coveted Grand Prix at the Festival of Empire Exhibition. This makes the ninth Grand Prix awarded to Oxo in the great International Exhibitions during the last four years.

### THE WEEK'S NOVELTIES.

### A NEW PATENT PHONOPHORE STETHOSCOPE.

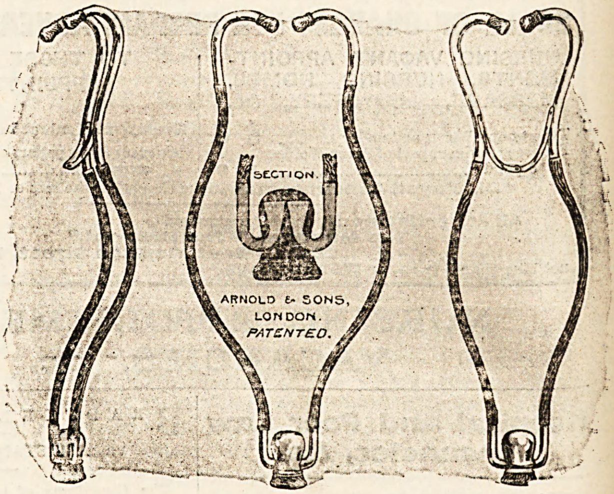

Messrs. Arnold and Sons, Giltspur Street. This new phonophore stethoscope is provided with two sound-tubes instead of one, as in the ordinary type—a very great improvement, as the sound travels through separate tubes direct to either ear instead of being divided at the top of the chest piece, which naturally prevents each ear obtaining equal sounds. Apart from this, the sound is greatly increased—a point which may appeal to many practitioners. The new stethoscope is particularly useful in cases of doubtful cardiac murmurs or where emphysematous lung renders the heart sounds almost inaudible. Most lung sounds are also well brought out by its use. Normal breath sounds are heard more loudly than with the ordinary stethoscope. The prices are the same as the old phonophore.

### APPOINTMENTS VACANT.

Full particulars of these vacancies can be obtained on reference to our advertisement columns:

Bradford Royal Infirmary.—House Physician.
Cornwall County Asylum, Bodmin.—Third Assistant Medical Officer.
Coventry and Warwickshire Hospital.—Junior House Surgeon.
Croydon General Hospital.—Junior House Surgeon.
Evelina Hospital, Southwark.—Clinical Assistants.
Leeds General Infirmary.—Resident Medical Officer.
Leith Hospital.—Outdoor Visiting Physician.
Parish of Liverpool.—Resident Assistant Medical Officers.
Royal Surrey County Hospital, Guildford.—Assistant House Surgeon.
Salford Royal Hospital.—Casualty House Surgeon.
Seamen's Hospital Society, Greenwich.—Two House Physicians, two House Surgeons, and Honorary Anæsthetist.
Warneford Hospital, Leamington.—House Surgeon.
Wolverhampton General Hospital.—House Surgeon.

### TO HOSPITAL SECRETARIES AND OTHERS.

We invite contributions from any of our readers, and shall be glad to pay, according to length, for items of news for the institutional section (including appointments, resignations, gifts, etc.) which we may publish. All payments for manuscripts used are made as early as possible after the beginning of each quarter.

